# Searching beyond the streetlight: Neonicotinoid exposure alters the neurogenomic state of worker honey bees

**DOI:** 10.1002/ece3.8480

**Published:** 2021-12-20

**Authors:** Nadejda Tsvetkov, Amro Zayed

**Affiliations:** ^1^ Department of Biology York University Toronto ON Canada

**Keywords:** agriculture, clothianidin, ecotoxicology, insects, transcriptomics

## Abstract

Neonicotinoid insecticides have been implicated in honey bee declines, with many studies showing that sublethal exposure impacts bee behaviors such as foraging, learning, and memory. Despite the large number of ecotoxicological studies carried out to date, most focus on a handful of worker phenotypes leading to a “streetlight effect” where the *a priori* choice of phenotypes to measure may influence the results and conclusions arising from the studies. This bias can be overcome with the use of toxicological transcriptomics, where changes in gene expression can provide a more objective view of how pesticides alter animal traits. Here, we used RNA sequencing to examine the changes in neurogenomic states of nurse and forager honey bees that were naturally exposed to neonicotinoids in the field and artificially exposed to neonicotinoids in a controlled experiment. We found that neonicotinoid exposure influenced the neurogenomic state of foragers and nurses in different ways; foragers experienced shifts in expression of genes involved in cognition and development, while nurses experienced shifts in expression of genes involved in metabolism. Our study suggests that neonicotinoids influence nurse and forager bees in a different manner. We also found no to minimal overlap in the differentially expressed genes in our study and in previously published studies, which might help reconcile the seemingly contradictory results often reported in the neonicotinoid literature.

## Graphical abstract

We used RNA sequencing to examine the changes in neurogenomic states of nurse and forager honey bees that were naturally exposed to neonicotinoids in the field and artificially exposed to a neonicotinoid in a controlled experiment. We found that neonicotinoid exposure influenced the neurogenomic state of foragers and nurses in vastly different ways. Additionally, we found no to minimal overlap in the differentially expressed genes in our study and in previously published studies, which demonstrates the importance of conducting honey bee research under field conditions.

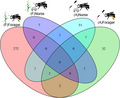

## 1 INTRODUCTION

There is a large body of literature showing sometimes contradicting effects of neonicotinoid (NNI) insecticides on the health of honey bees (Alkassab & Kirchner, 2017; Carreck & Ratnieks, 2014; Lawrence et al., 2016). The discrepancy in the literature has caused an academic debate regarding the impact of NNIs on bees and the possible biases that may lead to these inconsistencies (Benuszak et al., 2017; Blacquiere et al., 2012; Carreck & Ratnieks, 2014; Cutler & Scott‐Dupree, 2016; Cutler et al., 2014). Perhaps more importantly, it has also caused different regulatory agencies to reach contradicting conclusions regarding the safety of NNIs (Canada, 2017; Department for Environment, 2017).

Part of the discrepancy in the literature can be explained by basic study methodology (e.g., field vs. laboratory conditions; Carreck & Ratnieks, 2014), yearly fluctuations in colony phenotypes, even within the same study (Osterman et al., 2019), or interactions between NNI exposure and the local environment (Woodcock et al., 2017). Moreover, researchers typically focus their efforts on a few specific phenotypes to quantify, and this “observation bias” may influence the outcomes of toxicological studies. Sublethal effects, which are defined as physiological and/or behavioral effects after an exposure to a non‐lethal dose (Alkassab & Kirchner, 2017), by their definition, are endless in scope. Practically, researchers typically decide on one or two easily quantified phenotypes to study prior to toxicological testing, and such studies will miss any sublethal effects on unstudied phenotypes. Moreover, when researchers pick a phenotype to assess, they may introduce bias by choosing phenotypes that are a priori predicted to change (or perhaps not change) following exposure. These issues collectively lead to a “streetlight effect,” which occurs when people search for missing objects (knowledge in our case!) in the place where it is easiest to look—a problem that is common in many scientific fields (e.g., Battaglia & Atkinson, 2015).

One way to reduce the “streetlight effect” in ecotoxicological studies is to quantify a very large number of phenotypes—a type of inquiry that is suitable for the application of genomics. For example, RNA sequencing (RNAseq) provides a feasible and objective way to simultaneously query transcript abundance for thousands of genes and quantify how gene expression changes in response to pesticide exposure (Grozinger & Zayed, 2020; Lozier & Zayed, 2017). With this approach, the thousands of observed phenotypes (i.e., expression of thousands of genes) are chosen without any prior knowledge. Moreover, given the large body of knowledge linking genes with biological process and molecular functions in insects (Ashburner et al., 2000; Consortium, 2019; Kanehisa & Goto, 2000), it is possible to use transcriptomics to provide insight into the typical traits, and molecular and physiological processes that are impacted by exposure to pesticides and contaminants.

In honey bees, previous research has clearly demonstrated a very strong relationship between brain gene expression and typical worker behaviors (Zayed & Robinson, 2012). For example, to our knowledge, all distinct honey bee behavioral states studied to date (e.g., aggression, scouting, foraging, communication, and learning) appear to be associated with a specific pattern of differential gene expression (i.e., neurogenomic state) in the brain involving tens to thousands of genes (Wang et al., 2013; Zayed & Robinson, 2012). In many cases, this close relationship between neurogenomic state and behavioral state is causal, where shifts in neurogenomic states lead to shifts in worker behavior (Zayed & Robinson, 2012). As such, analysis of brain transcriptomes of bees exposed to pesticides is, in theory, capable of highlighting how pesticides impact bee behavior.

Although a few studies explored the effects of NNIs on the honey bee transcriptome (Christen et al., 2018; Li et al., 2019; Morfin et al., 2019; Wu, Luo, et al., 2017), none have done so for worker bees that were naturally exposed in the field. This is important because De Smet et al. (2017) demonstrated that there is a significant difference in gene expression response to NNIs between honey bees exposed in the hive versus those exposed in laboratory cages. Additionally, it is not clear how NNI exposure influences gene expression in the different honey bee adult worker castes, such as nurses and foragers (Winston, 1991).

We analyzed the brain transcriptomes of worker honey bees exposed to NNIs under field conditions and worker honey bees that were experimentally exposed through a clothianidin spiked pollen patty placed inside the hive (Tsvetkov et al., 2017). Briefly, we first conducted a season‐long field study that utilized colonies kept immediately near and at least 3 km away from corn in Canada to determine typical routes of exposure to NNIs. Corn production represents the largest use of arable land in North America (Hamel & Dorff, 2014), and almost all corn is grown from NNI‐treated seeds (Stewart & Baute, 2013). We collected honey bee foragers and nurses from these field colonies right after corn planting in late May for gene expression analysis, allowing us to explore how natural exposure to the myriads of agrochemicals, including NNIs, influences the typical neurogenomic state of worker bees. We then performed a controlled experiment mimicking the NNI exposure we found in the field (Tsvetkov et al., 2017) by feeding honey bee colonies clothianidin‐infused pollen patties. We collected foragers and nurses 30 days after the start of the experiment allowing us to investigate how chronic sublethal exposure to clothianidin influences the neurogenomic state of worker bees.

## 2 MATERIALS AND METHODS

### 2.1 Honey bees

**FIGURE 1:**
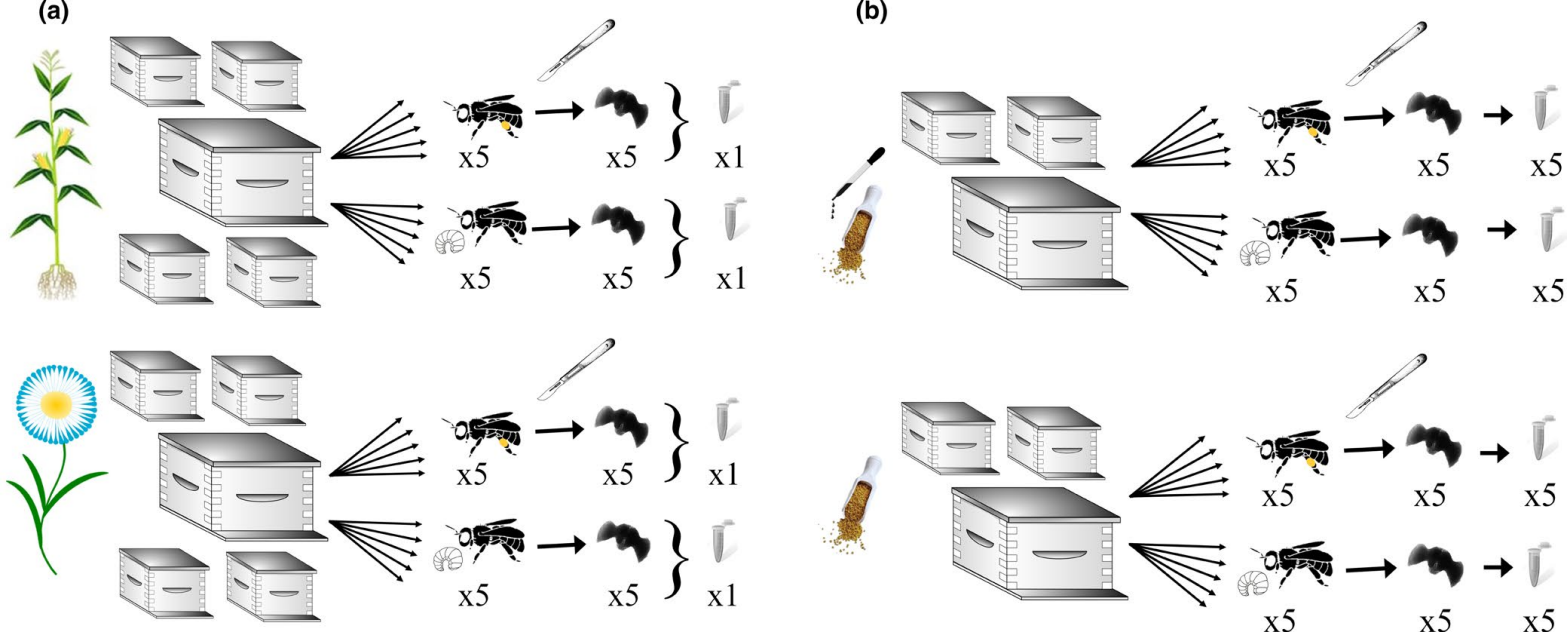
Study design. (a) Field Study—Five honey bee colonies were located near a neonicotinoid‐treated corn field, and five honey bee colonies were located at least 3 km away from agricultural fields. Five nurse bees and five forager bees were sampled from each colony. We dissected their brains and pooled five brains from each colony and each caste into a single sample for RNA extraction. (b) Apiary study—Three honey bee colonies received a pollen supplement infused with clothianidin, and three colonies received a control pollen supplement. Five nurse bees and five forager bees were sampled from each colony. We dissected their brains and extracted RNA from each of them separately

Honey bees were collected from colonies described in detail by Tsvetkov et al. (2017). Briefly, in the field study, 55 bee colonies were randomly allocated to five apiaries located near (<500 m) NNI‐treated corn (“exposed sites”) or to six apiaries located at least 3 km from agriculture (“unexposed sites”). For the current study (Figure 1), bees were collected from two different sites studied by (Tsvetkov et al., 2017), one near corn (Wellington 2) and one at least 3 km away from corn (Toronto 2). Each site had five colonies, and we collected five foragers and five nurses from each colony. We identified foragers as those bees returning to the hive with pollen loads and nurses as those bees who inserted their heads into a cell with larvae (Winston, 1991). The bees were collected right after corn planting on May 30, 2014 because of previous reports that corn planting coincided with bee mortality in Canada (Canada, 2012). The samples were transported to the laboratory on dry ice and later stored at −80°C until analysis. Pesticide analysis of the pollen from the Wellington county site “2” found 6.6 ppb of clothianidin and 2.0 ppb of thiamethoxam on May 5, 2014 and 11.5 ppb of clothianidin and 4.0 ppb of thiamethoxam on May 30, 2014 (Figure S1A; Tsvetkov et al., 2017). Pesticide analysis of the pollen in the colonies from the Toronto site “2” found no NNI residues in early or late May (clothianidin limit of detection [LOD]: 1.4 ppb; imidacloprid LOD: 0.7 ppb; thiamethoxam LOD: 0.7 ppb; acetamiprid LOD: 1.0 ppb; thiacloprid LOD: 0.9 ppb; nitenpyram LOD: 0.8 ppb; dinotefuran LOD: 0.6 ppb) (Tsvetkov et al., 2017). We also found coumaphos (miticide), carboxin (fungicide), imazlil (fungicide), and diuron (herbicide) in the Toronto “2” site and coumaphos and pyraclostrobin (fungicide) in the Wellington “2” site (Tsvetkov et al., 2017).

In 2015, we carried out an experiment mimicking the NNI field exposure found in 2014 (Tsvetkov et al., 2017). Ten colonies in a single apiary received artificial pollen for a 12‐week period. Half of the colonies received a pollen patty spiked with clothianidin, the most commonly found NNI in the field study. The exposure started with 4.9 ppb of clothianidin in week 1 and was lowered each week, until the dose reached 2.0 ppb at week 5 and stayed the same until week 12 (Figure S1B). Five nurses and five foragers were collected from each queen‐right colony 30 days after the start of our experiment. All of the bees were placed on dry ice immediately after collection and then stored at −80°C.

### 2.2 RNA extraction and analysis

We performed the brain dissections on dry ice in cold ethanol. The hypopharyngeal gland was removed as well as the ocelli. For the field bees, five nurse brains were pooled into one sample and five forager brains were pooled into another sample, while for the apiary bees, each brain was extracted and analyzed individually. We extracted RNA from the brains using RNeasy Mini Kit (Qiagen) following manufacturer instructions.

The samples were sent to Gѐnome Quѐbec's Innovation Center for stranded library preparations and illumina RNA sequencing with HiSeq4000 PE100, with 100 base pairs read length. We used Trimmomatic (Bolger et al., 2014) to remove adapters, low quality bases (leading and trailing 20), and low quality reads (reads shorter than 50 bases and when the average quality per base drops below 25 for a 20 base window). After filtering, the average across samples was 41.7 million sequences in 98.4 base pair length. Then, we used FastQC (Bioinformatics, 2011) to perform quality control checks on the following criteria: mean quality score, per sequence quality score, per base sequence content (ATGC), per sequence GC content, per base N content, sequence length distribution, and adapter content. The data successfully passed the quality check in all relevant areas.

The field and apiary studies were analyzed separately. At the time of bee collection, three exposed and three unexposed colonies were queen‐right, and a single sample of nurses from an unexposed field site was lost. Thus, the field study had a total of 19 pooled samples, and the apiary study had 60 samples. The RNA sequences were aligned and gene expression quantified with the amel 4.5 *Apis mellifera* genome assembly and Official Gene Set v3.2 (C. Elsik et al., 2014; Elsik et al., 2015; Weinstock et al., 2006) using Spliced Transcripts Alignment to a Reference (Dobin et al., 2013). The gene expression counts were then processed using EdgeR (McCarthy et al., 2012; Robinson et al., 2010) in R version 3.6.3 (Team, 2005). Any genes that had an expression count lower than 1 count per million (cpm) in less than two samples were filtered out. We detected the following number of expressed genes after filtering: field foragers: 10,976; field nurses: 10,852; apiary foragers: 11,381; and apiary nurses: 11, 271. The remaining counts were normalized using the default trimmed mean of M‐values method (McCarthy et al., 2012; Robinson et al., 2010). The dispersions of the models were estimated using the Cox–Reid profile‐adjusted likelihood method (McCarthy et al., 2012). Genes were denoted as differentially expressed if the *p*‐value was below .05 after a Benjamini adjustment (Benjamini & Hochberg, 1995).

### 2.3 Functional annotation analysis

In order to gain further insight into the function of the differentially expressed genes, we converted the honey bee genes into *Drosophila melanogaster* homologues using HymenopteraMine (Elsik et al., 2016). We mapped the following number of genes onto *D*. *melanogaster*: 2014 foragers: 7974; 2014 nurses: 7954; 2015 foragers: 8097; and 2015 nurses: 8075. Then, we analyzed the differentially expressed genes using DAVID 6.8 (Huang et al., 2008a, 2008b). We used the following options: Biological Process All, Molecular Function All, and Cellular Component All, as well as Kyoto Encyclopedia of Genes and Genomes (KEGG) Pathway analysis and Keywords. The *p*‐values for the enrichment analyses were corrected using the Benjamini adjustment (Benjamini & Hochberg, 1995). Illustrations of the enrichment analysis were performed using the online tool GOrilla (Eden et al., 2007, 2009) using the *D*. *melanogaster* homologues.

### 2.4 Comparisons

We compared our gene lists with previously published research on the effects of NNIs on honey bee gene expression (Christen et al., 2018; Derecka et al., 2013; Li et al., 2019; Morfin et al., 2019; Shi et al., 2017; Wu, Chang, et al., 2017; Wu, Luo, et al., 2017). Previously published differentially expressed gene lists were obtained from the supplementary data provided by the authors with each publication. Where required, the gene names were converted into the current iteration of the honey bee genome annotation using HymenopteraMine (Elsik et al., 2016). These gene lists were compared with the differentially expressed genes found in our study and with the background gene lists from our study. Then, a hypergeometric test (Johnson et al., 2005) was conducted in order to determine if the overlap was statistically different from chance and a *p*‐adjustment was done using the Holm–Bonferroni method (Holm, 1979). These tests were performed in R version 3.6.3 (Team, 2005).

## 3 RESULTS

### 3.1 Field study—foragers

We detected 278 differentially expressed genes (DEGs; Benjamini corrected *p* < .05) in the brains of foragers collected from colonies located near and far from NNI‐treated corn. Two hundred fifty one of them were upregulated in the field‐exposed foragers (Table S1). A GO analysis showed a significant enrichment of several biological processes, including “regulation of transcription” (Figure S2A, Benjamini corrected *p* < .05, Table S2), as well as an array of post‐embryonic development processes, including “tube development” (Figure S2B, Benjamini corrected *p* < .05, Table S2). We also examined if specific annotation clusters were enriched among DEGs and found that the top three clusters contained terms associated with “post‐embryonic development” (Enrichment score 8.35, Table S2), “tissue development” (Enrichment score 5.27, Table S2), and “brain development” (Enrichment score 3.93, Table S2). Other notable enriched clusters were “neuron development,” “learning and memory,” and “regulation of glucose metabolic process” (Table S2).

The GO analysis revealed an enrichment for regulation of transcription; thus, we compared our honey bee DEGs with the transcription factors identified in the honey bee transcriptional regulatory network discovered by Chandrasekaran et al. (2011). We found 16 overlapping transcriptional factors, which represented a statistically significant overlap (Table S3; hypergeometric test, *p* < .001). Twelve of these 16 transcription factors are involved in behavioral maturation of honey bee workers, while four were involved in foraging behaviors (Chandrasekaran et al., 2011). Two of the transcription factors were downregulated in foragers located near NNI‐treated corn. One was *kruppel homologue 1* (*Kr*‐*h1*) and the other *zinc finger protein 578* (*LOC411780*). Several detoxification genes were also differentially expressed. They were as follows: *CYP9Q1* (*LOC410492*; logFC: 0.70), *cytochrome P450 314A1* (*Cyp314a1*; also known as *shade*; logFC: 0.62), *carboxylesterase* (*LOC726134*; logFC: 1.10), and *NADPH*‐*cytochrome P450 reductase* (*LOC724870*; logFC = −0.45).

### 3.2 Field study—nurses

In contrast to the large number of DEGs found in foragers, we only detected nine differentially expressed genes (Benjamini corrected *p* < .05) in nurses, most of which (seven genes) where downregulated in the bees collected near NNI‐treated corn. A GO analysis revealed an enrichment of the annotation term “signal” (Benjamini corrected *p* < .008, Table S2). The KEGG pathway “starch and sucrose metabolism” was marginally enriched among the DEGs (Benjamini corrected *p* = .054, Table S2). The largest log fold change was observed for *alpha*‐*amylase* (*LOC406114*; logFC: −5.62), *chymotrypsin inhibitor* (*LOC725114*; logFC: −5.09), and *pancreatic triacylglycerol lipase* (*LOC551268*; logFC: −4.09). Out of the two upregulated genes found in the nurses collected near NNI‐treated corn, *cuticular protein 5* (*CRP5*) had the highest logFC change (1.90), while the other was uncharacterized (L*OC100578072*; logFC = 1.88).

### 3.3 Apiary experiment—foragers

We discovered 45 genes to be differentially expressed (Benjamini corrected *p* < .05) in the apiary foragers, of which 38 were upregulated in the clothianidin exposed bees relative to controls. A GO analysis of revealed no enriched processes after a Benjamini correction (*p* > .05; Table S2). *Myosin heavy chain*, *non*‐*muscle* (*LOC100576096*) had one of the largest fold changes (3.47) and was also differentially expressed in field foragers and apiary nurses. A transcription factor, *brachyury protein* (*LOC412976*) and *unconventional myosin*‐*IXb* (*LOC551706*), an orthologue of *dachs*, were also upregulated.

Only seven genes were downregulated in the clothianidin‐exposed foragers. The two most downregulated were *triacylglycerol lipase* (*LOC551268*; logFC: −2.70), which was also differentially expressed in apiary nurses, and *fatty acid hydroxylase domain*‐*containing protein 2* (*LOC409360*; logFC: −2.02). The other genes of note were *cyp6as5*, *NADPH oxidase 5*, and *fatty acid hydroxylase domain*‐*containing protein 2*‐*like* (*LOC727357*). All are involved in oxidoreductase, and all were downregulated.

### 3.4 Apiary experiment—nurses

We detected 63 DEGs, 41 of which were upregulated in the clothianidin‐exposed nurses relative to controls. A GO analysis showed a significant enrichment of the cellular component “extracellular region” (Benjamini corrected *p* = .005, Table S2) and the annotation term “signal” (Benjamini corrected *p* = .001, Table S2). No other terms were statistically significant after a Benjamini correction (*p* > .05; Figure S3; Table S2). Here, the highest log fold change (8.51) was for *cuticle protein 18*.*7* (*LOC725804*) and *AMP deaminase 2* (logFC: 6.26). One transcription factor was upregulated, a *homeobox protein Hox*‐*B1a* (*LOC724422)*. One of the most downregulated genes was *TOX high mobility group box family member 3*‐*like* (*LOC107965368*; logFC: −2.15).

### 3.5 Overlapping DEGs

**FIGURE 2:**
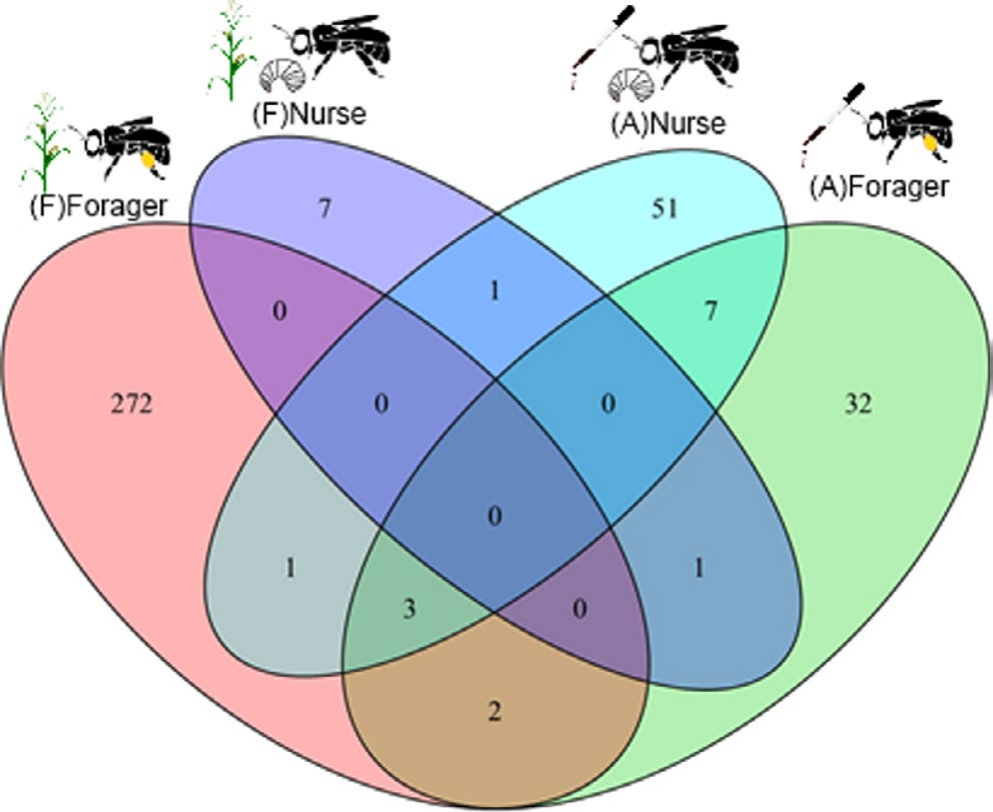
A Venn diagram of the number of overlapping differentially expressed genes between foragers collected in the field study [(F)Forager], nurses collected in the field study [(F)Nurse], foragers collected in the apiary experiment [(A)Forager], and nurses collected in the apiary experiment [(A)Nurse]. Statistically significant overlaps were found between all pairwise comparisons of these four groups (hypergeometric test, *p* < .05)

We compared the genes that were differentially expressed in our different experiments (Figure 2; Table S4). We found a statistically significant overlap between the DEGs found in the field‐exposed bees and in the apiary‐exposed bees (forager DEGs: five overlapped, hypergeometric test, *p* = .005; nurse DEGs: one overlapped, hypergeometric test, *p* = .049); these genes may be associated with NNI‐related exposure. We also found a statistically significant overlap between the DEGs found in the apiary‐exposed nurses and foragers (10 DEGs overlapped, hypergeometric test, *p* < .001), suggesting that there may be a common set of genes associated with clothianidin exposure in both castes. The DEGs found in the field‐exposed foragers and nurses did not overlap.

We compared our data with previously published studies on the transcriptome response following NNI exposure (Table 1). After a Holm–Bonferroni correction of the *p*‐value (Holm, 1979), we found a statistically significant overlap between the DEGs of our field‐exposed nurses and the DEGs reported by Christen et al. (2018) for imidacloprid (3 ppb: hypergeometric test, Holm–Bonferroni *p* = .003; 30 ppb: hypergeometric test, *p* = .004), and thiamethoxam (1 ppb: hypergeometric test, *p* = .007; 10 ppb: hypergeometric test, *p* < .001). We also found a statistically significant overlap between the DEGs found in our field‐exposed foragers and the DEGs found by Christen et al. (2018) in the 30 ppb imidacloprid treatment group (hypergeometric test, *p* = .006). Additionally, a statistically significant overlap was found between the DEGs of our field‐exposed nurses and those reported by Wu, Luo, et al. (2017) (hypergeometric test, *p* < .001). We found no statistically significant overlap between the DEGs in our study and those in the clothianidin treatment group reported by Christen et al. (2018) (3 ppb: 0 DEGs, 30 ppb: hypergeometric test, *p* = .0869). Likewise, we found no statistically significant overlap between the DEGs of our study and those reported by Shi et al. (2017), those reported by Li et al. (2019), and those reported by Morfin et al. (2019) (hypergeometric test, *p* > .1).

**TABLE 1:** Summary of previously published papers on the effects of NNIs on the honey bee transcriptomes and the number of differentially expressed genes (DEGs) overlapping with the current study

Reference	Study design	Exposure duration	Dose and NNI	Sample analyzed	DEGs	Overlapping DEGs			
						Field forager (278)	field nurse (9)	apiary forager (45)	Apiary nurse (63)
Shi et al. (2017)	4‐day‐old workers taken from the hive and exposed in cages	10 days	10 ppb of TMX	Whole bees	609	9	2	3	7
Wu, Luo, et al. (2017)	Newly emerged workers raised in cages for one week and then exposed in cages	Up to 8 days	10 ppb of IMD	Pools of whole bees collected after 1, 2, 4, and 8 days of exposure	509	6	4*	2	3
Christen et al. (2018)	Mixed aged bees collected from the hive and exposed in cages	48 h	3 ppb of CLO	Bee brains and HPG	18	0	0	0	0
			30 ppb of CLO		244	6	1	2	1
			3 ppb of IMD	Bee brains and HPG	26	1	2*	1	0
			30 ppb of IMD		113	7*	2*	0	0
			1 ppb of TMX	Bee brains and HPG	6	0	1*	0	0
			10 ppb of TMX		25	1	2*	0	0
Morfin et al. (2019)	Newly emerged bees fed in cages	7 days	10 ppb of CLO	Bee brains	298	1	2	1	3
Li et al. (2019)	11‐day‐old workers taken from the hive and then exposed in cages	11 days	20 ppb of IMD	Bee brains	131	6	0	0	2
Derecka et al. (2013)	Colonies received sugar syrup with NNI	15 days	2 ppb of IMD	6–9‐day‐old larvae	300	3	4*	1	4
Wu, Chang, et al. (2017)	NNI solution was pipetted into the larvae cell in a colony	4 days	500 ppb of IMD	Bee heads from newly emerged bees	578	13	4*	4	4

Note

Statistically significant overlaps are marked with * and bolded (hypergeometric test, *p* < .05). The numbers denoted in the parenthesis are the number of DEGs found in the current study.

Abbreviations: CLO, clothianidin; HPG, hypopharyngeal gland; IMD, imidacloprid; NNI, neonicotinoid; TMX, thiamethoxam.

We also compared our study with the transcriptome studies that exposed larvae to NNIs (Table 1). We found a statistically significant overlap between our field‐exposed nurse DEGs and the DEGs reported by Wu, Chang, et al. (2017) and Derecka et al. (2013). No other overlap was statistically significant (hypergeometric test, *p* > .1).

## 4 DISCUSSION

In our study, we sought to use a transcriptomic analysis to provide an unbiased snapshot of the effects of field realistic exposure to NNIs on honey bees. First, we performed RNAseq analysis on the brains of foragers and nurses that were collected from colonies located near NNI‐treated corn fields and away from such fields. Second, we performed a similar transcriptomic analysis on the brains of foragers and nurses collected from colonies that were experimentally exposed to field realistic levels of clothianidin spiked into pollen patties relative to a control group. Across both the natural and experimental exposure, the neurogenomic states of exposed bees were clearly distinct from those of the unexposed bees. Our study unequivocally shows that exposure to sublethal doses of NNIs alters the neurogenomic states of forager and nurse honey bees in a distinct manner.

In honey bees, individual neurogenomic states are associated with many behaviors that impact colony fitness, such as aggression, scouting, foraging, communication (Zayed & Robinson, 2012), and learning and memory (Wang et al., 2013). This close relationship between brain gene expression and behavior is sometimes causal (Zayed & Robinson, 2012), suggesting that NNI‐induced changes to the neurogenomic state of exposed bees can potentially influence important behaviors relating to behavioral maturation, learning and memory, and foraging. Indeed, previous studies have demonstrated that exposure to NNIs can alter learning and memory (Tison et al., 2019; Zhang & Nieh, 2015) and navigation in honey bees (Fischer et al., 2014).

One important finding of our study is that nurses and foragers appear to respond differently to NNIs. There was no overlap in DEGs in the field‐exposed nurses and foragers and less than one fifth of DEGs overlapped between the two castes in the apiary bees. It is possible that this is due to the nurses and foragers experiencing different exposure to NNIs. However, given that there is a statistically significant overlap between nurses in the field and apiary and between foragers in the field and apiary, we believe that behavioral state (nurse vs. forager) is a major factor in how gene expression changes after NNI exposure. This indicates that it is crucial for any phenotypic assay to distinguish between these two worker castes, which contribute to colony fitness in different ways. The field RNAseq study strongly suggests that exposure influences phenotypes that are critical for resource gathering such as learning and memory, locomotion, and vision in foragers and phenotypes associated with carbohydrate metabolism in nurses. The latter may impact the ability of nurses to produce high quality brood food (Eischen et al., 1984).

We believe that our data show that field realistic exposure to NNIs affects the developmental process, which manifests itself in different manners during the life cycle of the bee. We found unconventional myosin‐IXb (*LOC551706*) to be upregulated in the exposed foragers in both the field and apiary studies. It is an orthologue to *dachs* in *D*. *melanogaster*, which influences growth through interactions with *Warts*, positive regulation of hippo signaling, and participates in Dachsous–Fat signaling (Misra and Irvine, 2016; Saavedra et al., 2016; Zhang et al., 2016). *LOC100576096* was upregulated in exposed foragers in both studies and in nurses in the apiary. It is described as myosin heavy chain, non‐muscle‐like, which is required for morphogenesis and cytokinesis (Sechi et al., 2014).

Our field‐exposed foragers had 16 transcription factors that were differentially expressed. Most of these transcription factors are known to play a role in the maturation process as bees transition from nursing to foraging (Chandrasekaran et al., 2011). NNIs have been shown to accelerate the maturation process and induce precocious foraging (Colin et al., 2019). Half of the transcription factors are also known to be involved in neural development or differentiation (Table S3). NNIs are known to affect learning and memory in honey bees (e.g., Tison et al., 2019) as well as navigation (Fischer et al., 2014; Tison et al., 2016).

Two of the transcription factors were downregulated in the field‐exposed foragers: *Kr*‐*h1* and *LOC411780*. *LOC411780* is a homologue to *D*. *melanogaster's crol*, which links the Ecdysone steroid hormone pathway and the Wingless signaling pathway. It is required for cell cycle progression, and reducing *crol* expression reduces wing size (Mitchell et al., 2008). *Kr*‐*h1* levels are higher in foragers when compared with nurse bees (Grozinger & Robinson, 2007), and *Kr*‐*h1* levels seem to be related to stable physiological changes that occur during the nurse–forager transition (Fussnecker & Grozinger, 2008).

Genes involved in starch and sucrose metabolism also significantly overlapped between our field‐exposed nurses and adult bees exposed to NNIs by Christen et al. (2018) and Wu, Luo, et al. (2017). Although few genes overlapped between the different groups in our study, GO terms related to glucose metabolism were found in our field‐exposed foragers and GO terms related to amino‐sugar metabolism were found in the apiary‐exposed foragers and nurses. This overlap, despite the varied exposure methods used in the studies, strongly suggests that these metabolic processes are part of the honey bees’ physiological response to NNIs.

A sizable proportion (113/395) of our DEGs had no orthologues or homologues in the fruit fly, which limits our ability to study the molecular functional and biological processes associated with such genes. Although it limits the current functional analysis, the lack of *D*. *melanogaster* homologues indicates that our DEGs contain a large number of taxonomically restricted genes, which tend to be associated with social behaviors of worker honey bees (Johnson & Tsutsui, 2011). It is thus possible that NNI exposure may specifically target genes involved in regulating social behavior in honey bees. Indeed, a recent study found that NNI exposure impairs nursing and alters social dynamics within the nest (Crall et al., 2018).

We found no statistically significant overlap between the DEGs found in our study and those found in three of the seven previously published transcriptomic studies (Li et al., 2019; Morfin et al., 2019; Shi et al., 2017). Some of the discrepancy can be explained by the fact that our field‐exposed bees were exposed to a cocktail of pesticides and thus are unlikely to exhibit the same gene expression response as bees exposed to single compounds (Christen et al., 2017). However, in the apiary study, we exposed our bees to a single NNI, and we did find a significant overlap between our field‐exposed bees and our apiary‐exposed bees, even though the chemical exposure profiles were not the same. In addition, the DEGs we found in our apiary‐exposed nurses, and foragers did not significantly overlap with any of the seven previously published transcriptome studies. We note that overlap in differentially expressed genes between different transcriptomic studies is the norm (Doublet et al., 2017; Guoth et al., 2020; Sobotka et al., 2016; Tsvetkov et al., 2021; Zayed et al., 2012; Zayed & Robinson, 2012). The lack of overlap between our study and others may have been caused by several reasons, including the following: (1) different cohorts of bees chosen for RNAseq analysis, (2) different exposure parameters and compounds, (3) different genetic backgrounds, and (4) cage versus field conditions.

One common problem of performing field studies is the possibility of contamination of the unexposed sites (Tsvetkov et al., 2017). One way we tried to overcome this is by testing for 231 different agrochemicals in six different matrices (pollen, nectar, nurse bees, forager bees, larvae, and dead bees), seven times over the active bee season in Ontario—from early May to late September. Our unexposed site had no detectable levels of NNIs in any of the matrices in any of the time points we sampled (Tsvetkov et al., 2017). It is possible, however, that there were NNIs present below the limits of detection. The limits of detection for NNIs ranged from 0.3 to 1.4 ppb depending on the matrix and particular NNI. Our “unexposed” field bees *may* have experienced small but non‐detectable doses of NNIs. While we can never be certain if the unexposed sites were indeed NNI‐free, our toxicological testing does indicate that our comparison between exposed and unexposed sites still reflect a big difference in the NNI residue experienced by colonies in these two sites in our experiment.

We investigated the effects of field realistic NNI exposure on global gene expression in the honey bee brain. We found that the DEGs are associated with development, particularly in bees exposed under field conditions. We also found no to minimal overlap in the DEGs in our study and in previously published studies, which further demonstrates the importance of conducting honey bee research under colony conditions. Our study offers novel insights into the effects of filed realistic NNI exposure on honey bee nurse and forager gene expressions. Future research could build on this work by exploring the effects of NNIs on metabolic pathways in nurses and on the effects of NNIs on the maturation process in honey bee workers.

## CONFLICT OF INTEREST

None declared.

## AUTHOR CONTRIBUTIONS

**Nadejda Tsvetkov:** Conceptualization (equal); Data curation (lead); Formal analysis (lead); Investigation (lead); Methodology (lead); Validation (lead); Visualization (lead); Writing – original draft (lead); Writing – review & editing (lead). **Amro Zayed:** Conceptualization (equal); Funding acquisition (lead); Writing – review & editing (equal).

## Supporting information

Supplementary Material

Figure S2A

Figure S2B

Table S1

Table S2

Table S5

## Data Availability

The data discussed in this publication have been deposited in NCBI's Gene Expression Omnibus (Edgar et al., 2002) and are accessible through GEO Series accession number GSE178742 (https://www.ncbi.nlm.nih.gov/geo/query/acc.cgi?acc=GSE178742).
