# Supplementary material for: Searching beyond the streetlight: Neonicotinoid exposure alters the neurogenomic state of worker honey bees
Source: Ecol Evol. 2021 Dec 20;11(24):18733–42. doi: 10.1002/ece3.8480 (PMC8717355; doi:10.1002/ece3.8480)
Supplement: Supplementary file 1 — Supplementary Material [file ECE3-11-18733-s003.docx]

(Separate File)

Supp. Table 1. Differentially expressed genes found in the brains of 2014 field exposed foragers and nurses and 2015 apairy exposed foragers and nurses.

(Separate File)

Supp. Table 2. The functinal anotation analysis of the *Drosophila melanogaster* homologues of the differentially exposed genes found in the brains of 2014 field exposed foragers and nurses and 2015 apairy exposed foragers and nurses.

Supp. Table 3. Differentially expressed transcription factors found in the 2014 filed exposed foragers. Fly (*Drosophila melanogaster)* homologues and their summaries from FlyBase (version FB2020_03) ([Thurmond et al., 2019](#_ENREF_1)).

| Supp. Table 3. Differentially expressed transcription factors found in the 2014 filed exposed foragers. Fly (*Drosophila melanogaster)* homologues and their summaries from FlyBase (version FB2020_03) ([Thurmond et al., 2019](#_ENREF_1)). | | | | |
| --- | --- | --- | --- | --- |
| **Gene ID** | **Symbol** | **logFC** | **Names** | **Fly homologue and its summary** |
| GB43022 | Kr-h1 | -0.603745059 | kruppel homolog 1 | Kruppel homolog 1 (Kr-h1) encodes a transcriptional regulator involved in axon pathfinding, neurite and axon remodeling as well as pupal photoreceptor maturation. In all cases, the function of the product of Kr-h1 is linked to that of the 20-hydroxyecdysone hormone |
| GB49050 | LOC411780 | -0.378313001 | zinc finger protein 578 | crooked legs (crol) encodes a zinc finger transcription factor induced by 20-hydroxyecdysone at the onset of metamorphosis. It regulates wg transcription and cell cycle progression in the wing. Its over-expression in the eye accelerates the cell cycle and de-represses silenced genes. |
| GB45157 | LOC410347 | 0.525648008 | protein big brother | Big brother (Bgb) encodes a beta-subunit of the transcription factor complex core binding factor, which is involved in transcription regulation. It regulates hemocyte proliferation and acts redundantly with the product of Bro in embryonic segmentation |
| GB49611 | LOC724983 | 0.550615975 | retinal homeobox protein Rx1 | Retinal Homeobox (Rx) encodes a homeodomain transcription factor required for processes of brain development. It is involved in growth regulation, proliferation and cell survival. |
| GB52109 | LOC726709 | 0.555363594 | uncharacterized LOC726709 | klarsicht (klar) encodes a member of the Nesprin family that links microtubule motors and various cellular structures. It controls the migration and positioning of nuclei in photoreceptors and muscles. It also regulates the motion of RNP granules in oocytes and lipid droplets in embryos. |
| GB44976 | LOC410253 | 0.616510546 | ataxin-2 homolog | vrille (vri) encodes a bZIP transcription factor acting as an enhancer of decapentaplegic phenotypes both in embryo and in wing. It is involved in hair and cell growth and in tracheal development. Vri is a clock-controlled gene acting as a repressor of the products of Clk and cry. |
| GB40150 | Foxp | 0.6255159 | FoxP protein | Forkhead box P (FoxP) encodes a transcription factor expressed in the nervous system. It is involved in locomotion, operant self-learning and courtship behavior. |
| GB47799 | LOC410468 | 0.633401237 | protein hairy | Flybase: hairy ([h](http://flybase.org/search/h)) encodes a bHLH transcriptional repressor that recruits the corepressor encoded by [gro](http://flybase.org/search/gro) to target promoters. It is a pair-rule gene that contributes to embryonic segmentation and peripheral neurogenesis. |
| GB47057 | LOC113218618 | 0.645198198 | protein tramtrack, beta isoform-like | tramtrack (ttk) - zinc finger - represses neural cell fate in the peripheral nervous system - a master repressor of enteroendocrine cell specification in intestinal stem cell lineages - regulates morphogenetic events during tracheal development. *tramtrack* is expressed downstream of Notch in the peripheral nervous system. |
| GB55387 | LOC100577139 | 0.66540567 | Lilliputian | Lilliputian (lilli) - transcription factor - Fragile X mental retardation 2 (Fmr2) family - Functions in MAPK and Dpp signaling pathways - affects growth, a function associated with the insulin pathway - affects the cytoskeleton early in development. |
| GB49751 | LOC551364 | 0.708469925 | homeobox protein SIX3 | Optix ([Optix](http://flybase.org/search/Optix)) encodes a homeobox containing DNA binding protein and a member of the SIX class of proteins. It functions as a repressor via interaction with the transcriptional co-repressor encoded by [gro](http://flybase.org/search/gro). It is involved in eye formation and morphogenetic furrow movement. |
| GB50686 | LOC408411 | 0.783927745 | transcription factor Sox-2 | SoxNeuro (SoxN) encodes an HMG-domain transcription factor. In early embryos it specifies neural progenitors in the central nervous system, while in later embryos it negatively regulates Wg signaling and controls expression of genes required for denticle construction with the product of ovo. |
| GB45063 | LOC726415 | 0.788613147 | LIM/homeobox protein Lhx9 | tailup (tup) encodes a transcription factor that regulates neuronal sub-type identity, including motor, serotonergic and dopaminergic neuron identity. It regulates germ band retraction, dorsal closure, muscle and heart development. |
| GB44042 | LOC411009 | 0.860651414 | dachshund homolog 2 | dachshund ([dac](http://flybase.org/search/dac)) encodes a transcriptional cofactor that physically interacts with several other retinal determination proteins, including those encoded by [eya](http://flybase.org/search/eya) and [so](http://flybase.org/search/so), and regulates eye, leg, gonad, and brain development |
| GB50048 | Mblk-1 | 0.881577442 | transcription factor mblk-1-like | Ecdysone-induced protein 93F (Eip93F) - Rho-type guanine nucleotide exchange factor (Eip93F) encodes a DNA binding protein that plays an >> important role as an adult determinant during fly metamorphosis. |
| GB44229 | LOC724740 | 0.908857295 | fork head domain transcription factor slp2 | sloppy paired 2 (slp2) encodes a transcription factor of the fork-head family. Together with the product of slp1, it regulates a wide variety of developmental processes including embryonic segmentation, ventral fate specification in the retina, and temporal patterning of the neuroblasts that produce medulla neurons. |

| Supp. Table 4. The overlapping DEGs between the different groups within this study. F2014 stands for field exposed foragers in 2014; N2014 stands for field-exposed nurses in 2014; F2015 stands for apiary exposed foragers in 2015; N2015 stands for apiary exposed nurses in 2015 | | | | | |
| --- | --- | --- | --- | --- | --- |
| **F2014** | **N2014** | **F2015** | **N2015** | **Symbol** | **Description** |
| + | - | + | + | LOC102654154 | uncharacterized LOC102654154 |
| + | - | + | + | LOC100576096 | myosin heavy chain, non-muscle |
| + | - | + | + | LOC102656247 | dynein-1-beta heavy chain, flagellar inner arm I1 complex |
| + | - | + | - | LOC551782 | bestrophin-4 |
| + | - | + | - | LOC551706 | unconventional myosin-IXb |
| + | - | - | + | LOC113218918 | putative mediator of RNA polymerase II transcription subunit 26 |
| - | - | + | + | LOC413693 | cilia- and flagella-associated protein 251 |
| - | - | + | + | LOC726981 | circadian clock-controlled protein |
| - | - | + | + | LOC100576287 | uncharacterized LOC100576287 |
| - | - | + | + | LOC551347 | UPF0605 protein CG18335 |
| - | - | + | + | LOC410606 | protein Skeletor, isoforms B/C |
| - | - | + | + | LOC411651 | Bardet-Biedl syndrome 7 protein homolog |
| - | - | + | + | LOC100577541 | uncharacterized LOC100577541 |
| - | + | + | - | LOC551268 | pancreatic triacylglycerol lipase |
| - | + | - | + | CPR5 | cuticular protein 5 |

(Separate File)

Supp. Table 5 - Summary of previously published papers on the effects of NNIs on the honey bee transcriptomes and the number of differentially expressed genes (DEGs) overlapping with the current study. Additional information regarding maximal DEGs overlapped due to chance.

(Separate File) Supp. Fig. 2. Visualization of the functional analysis of the differentially expressed *drosophila melanogaster* homologues in the 2014 field exposed foragers. Separated into (**A**) “biological regulation” and (**B**) “Others” for clarity. Colored cells represent biological processes that were statistically significantly enriched after a Benjamini correction. The darker the color the lower the p value.

Supp. Fig. 1. The amount of neonicotinoids found in (**A**) the pollen of the colonies located near corn in the 2014 field study and (**B**) in the pollen supplements given to the exposed colonies in the 2015 Apiary Experiment. Bees for the RNA analysis were collected on May 30^th^ in 2014 and at day 30 from the start of the experiment in 2015. ([Tsvetkov et al., 2017](#_ENREF_2))

Sup. Fig. 3. Visualization of the functional analysis of the differentially expressed *drosophila melanogaster* homologues in the 2015 apiary exposed nurses. Colored cells represent terms that appeared in analysis. None of the terms were statistically significant after a Benjamini correction; however the molecular function of ‘chitin binding’ was statistically enriched in the honey bee gene analysis (p=0.019, Sup. Table 2).


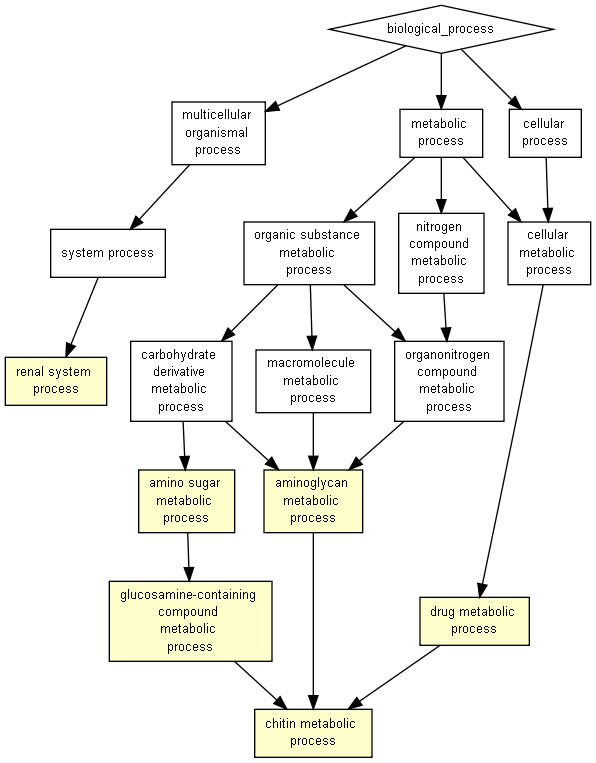


**References**

Thurmond, J., Goodman, J. L., Strelets, V. B., Attrill, H., Gramates, L. S., Marygold, S. J., . . . Trovisco, V. (2019). FlyBase 2.0: the next generation. *Nucleic acids research, 47*(D1), D759-D765.

Tsvetkov, N., Samson-Robert, O., Sood, K., Patel, H., Malena, D., Gajiwala, P., . . . Zayed, A. (2017). Chronic exposure to neonicotinoids reduces honey bee health near corn crops. *Science, 356*(6345), 1395-1397.
