# Supplementary figures and images for: Searching beyond the streetlight: Neonicotinoid exposure alters the neurogenomic state of worker honey bees
Source: Ecol Evol. 2021 Dec 20;11(24):18733–42. doi: 10.1002/ece3.8480 (PMC8717355; doi:10.1002/ece3.8480)

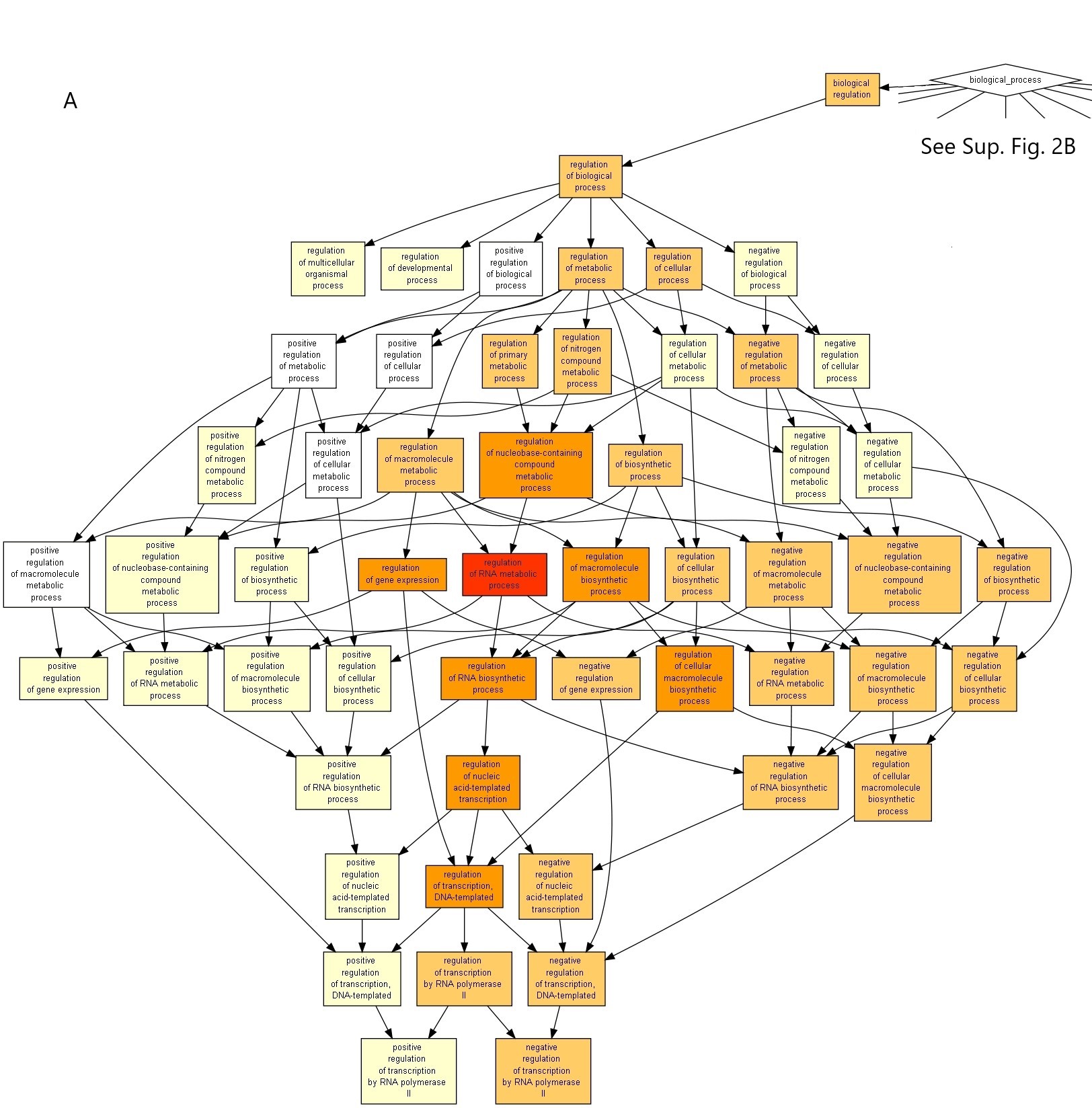

Supplement: Supplementary file 2 — Figure S2A [file ECE3-11-18733-s002.jpg]

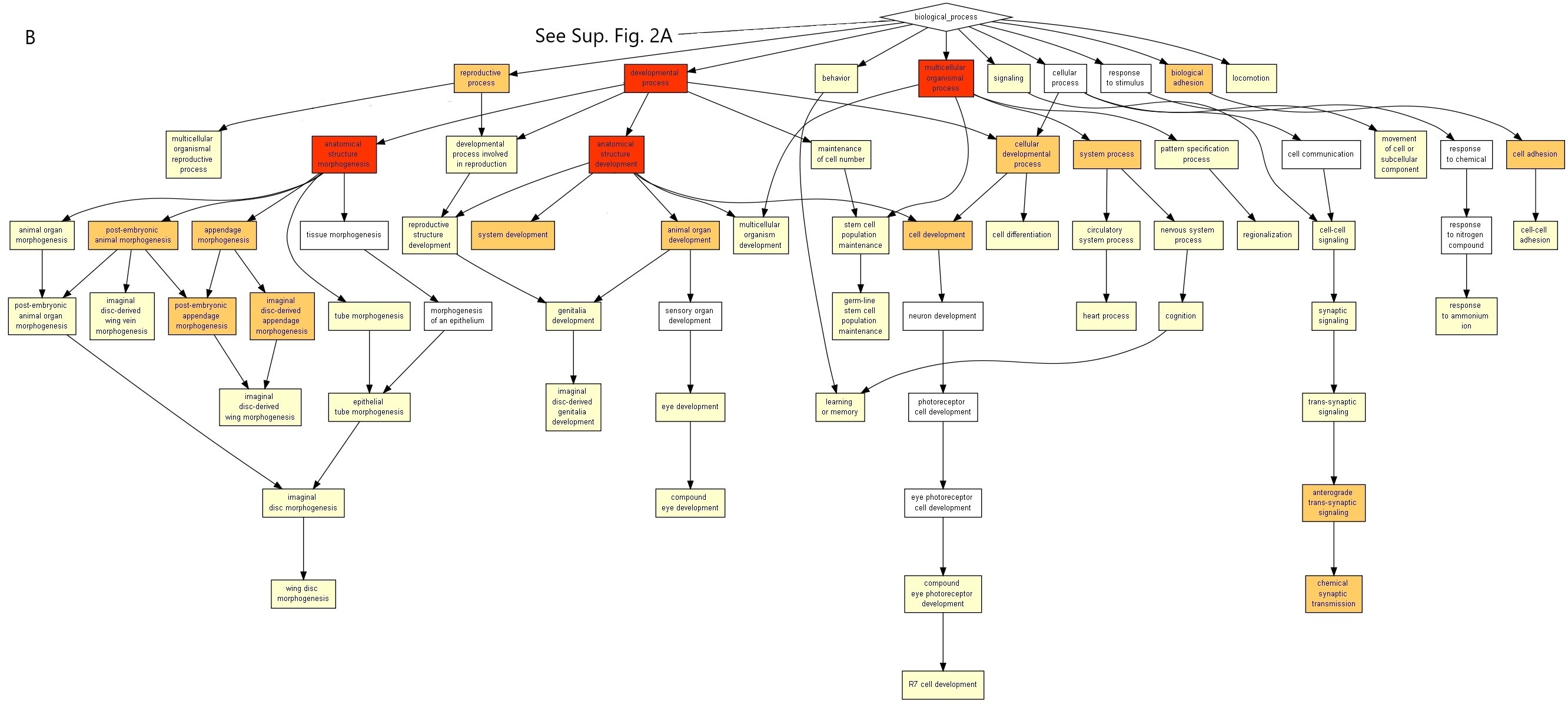

Supplement: Supplementary file 3 — Figure S2B [file ECE3-11-18733-s001.jpg]
